# Energy-Efficient, On-Demand Activation of Biosensor Arrays for Long-Term Continuous Health Monitoring

**DOI:** 10.3390/bios12050358

**Published:** 2022-05-21

**Authors:** Jonathan Lundquist, Benjamin Horstmann, Dmitry Pestov, Umit Ozgur, Vitaliy Avrutin, Erdem Topsakal

**Affiliations:** 1 Department of Electrical and Computer Engineering, College of Engineering, Virginia Commonwealth University, 907 Floyd Ave, Richmond, VA 23284, USA; lundquistjd@vcu.edu (J.L.); horstmannbm@vcu.edu (B.H.); uozgur@vcu.edu (U.O.); etopsakal@vcu.edu (E.T.); 2 Nanomaterials Core Characterization Facility, College of Engineering, Virginia Commonwealth University, 907 Floyd Ave, Richmond, VA 23284, USA; dpestov@vcu.edu

**Keywords:** biosensor array, pulse current activation, continuous glucose monitoring

## Abstract

Wearable biosensors for continuous health monitoring, particularly those used for glucose detection, have a limited operational lifetime due to biodegradation and fouling. As a result, patients must change sensors frequently, increasing cost and patient discomfort. Arrays of multiple sensors, where the individual devices can be activated on demand, increase overall operational longevity, thereby reducing cost and improving patient outcomes. This work demonstrates the feasibility of this approach via decomposition of combustible nitrocellulose membranes that protect the individual sensors from exposure to bioanalytes using a current pulse. Metal contacts, connected by graphene-loaded PEDOT:PSS polymer on the surface of the membrane, deliver the required energy to decompose the membrane. Nitrocellulose membranes with a thickness of less than 1 µm consistently transfer on to polydimethylsiloxane (PDMS) wells. An electrical energy as low as 68 mJ has been shown to suffice for membrane decomposition.

## 1. Introduction

There is currently a strong demand for long-term continuous health monitoring of human physiology, driven by a desire to refocus care from a reactive model to a preventative one. Physiological parameters such as pulse rate, oxygen saturation, sleep disturbances, body temperature, and daily physical activity are beamed right into our personal cloud of data via smart watches [1], but measuring other physiological parameters requires more sophisticated devices. The next generation of sensors incorporates aspects of AI, IoT, and 5G communications to provide more detailed data to healthcare providers in real time, expanding the possibilities of point-of-care testing [2]. The level of personalized care these sensors can provide has the potential to significantly enhance the quality of life for patients and providers managing long-term illnesses [3]. In the future, these sensors may provide critical patient data to providers to assist in the care of long-term diseases such as Parkinson’s and diabetes [4] as well as generalized health monitoring [2,3].

Sensors capable of detecting and monitoring a range of conditions [5,6,7] form a new tool in our healthcare arsenal that enormously boosts early diagnostic capabilities and enables active management of personal health by improving lifestyle. Effective ways to monitor physiologically significant parameters involve sampling biological fluids such as saliva, sweat, blood, or urine. While blood typically offers the highest concentrations and ranges of relevant biomarkers, sample collection (patient compliance) and storage are often challenging. Monitoring interstitial fluid (ISF) through subcutaneous biosensors offers an attractive alternative, with less concerns associated with device fouling that blood contacting devices are prone to. ISF levels of low molecular weight analytes (glucose, L-lactate, uric acid, folic acid, etc.) tend to match blood biomarker levels closely [8] with short lag times [9,10], (1–3 and 5–10 min for glucose level in the dermis and the adipose tissue, respectively [11]).

The most common biosensors are electrochemical sensors, which are more specific and efficient than devices based on noninvasive techniques such as nuclear magnetic resonance (NMR) spectroscopy [12], radioisotope tracing [13,14], and microfluorometric assays [15]. The advantages of electrochemical sensors include real-time monitoring capability, ease of fabrication and control, reproducibility, and low cost. High sensitivity and wide detection range of electrochemical biosensors have been demonstrated for a variety of physiologically important analytes present in ISF (glucose [16,17,18], cholesterol [19,20], uric [21,22], and L-lactic [23,24], acids, potassium [25], sodium [26], calcium [27,28], etc.). Unfortunately, current state-of-the-art implantable electrochemical glucose sensors (produced by Medtronic PLC, Dublin, Ireland, Abbott, Chicago, IL, USA, or Dexcom, San Diego, CA, USA) have functional longevity limited to 2 weeks at best due to degradation of working electrodes and fouling from fibrosis and inflammation [29,30], not to mention their relatively large size. Longevity has been extended to 90 days by a recently introduced implantable optical sensor, but its sensitivity still needs improvement in the hypoglycemic range [31].

The best example of a continuous electrochemical sensor on the market today is continuous glucose monitoring (CGM), which has been shown to improve patient outcomes by aiding in glycemic control and patient compliance [32]. Furthermore, there is evidence that CGM reduces patient anxiety, which may lead to better quality of life [33]. Even with these advantages, there are still hurdles for adoption of CGM. Patients often find CGMs expensive when compared with other methods of monitoring blood glucose [34]. One method to reduce the cost of the system would be to find a way to extend the operational lifetime of these devices. Cost and lifetime limitations apply not only to CGMs, but to any continuous health monitor that is based on electrochemical sensing or that is in direct contact with blood or bodily fluids [35]. A possible solution to this longevity problem, is using protective membranes isolating individual sensors in a sensor array so that each sensor can be sequentially and rapidly activated through electrical rupture of the protective membrane at a desired time before the previous sensor fails. Such an approach enables continuous monitoring of analytes for an extended time, determined by the number of sensors in the array. The array can also be composed of different types of biosensors targeting different analytes. Activation could be achieved through the use of membranes based on an energetic polymer such as nitrocellulose being heated with a resistive filament using a current pulse [36,37,38]. The filament is used to concentrate power loss of the applied current pulse directly on the membrane, thereby igniting the nitrocellulose and disintegrating the membrane that is separating the biofluid from the sensor being placed online. Nitrocellulose, already commonly used in biosciences for protein blotting [39] can also be formed by spin coating and has a low decomposition temperature, with self-decomposition starting at 55 °C and fast self-destruction at 130 °C [40]. This study focused on developing an efficient method of nitrocellulose membrane fabrication and transfer to polydimethylsiloxane (PDMS) wells that house biosensors, while also producing an energy-efficient, mechanically robust, and cost-effective on-demand biosensor activation mechanism. Careful selection of spin-coating substrate for membrane fabrication and materials used to fabricate the filaments resulted in a cost-effective sensing well that could be activated with as little as 68 mJ of energy. This low energy may lead to circuitry that could be miniaturized for use with implanted or on-body health monitoring devices.

## 2. Materials and Methods

In this study, polymer membranes containing an oxidizing nitro-group (nitrocellulose, a material known as an energetic polymer) are employed to prevent biosensors from premature exposure to interstitial fluid (ISF). A thin filament was placed on the membrane surface. Membrane decomposition was triggered by sending a short pulse of electrical current. The use of oxidizing groups allows a substantial decrease in the decomposition threshold temperature, and therefore, the electrical current required to disintegrate the membrane. The effect of filament design, at submicron membrane thickness on disintegration threshold energy is explored.

The construction process for the pulse current activated sensing well arrays consists of four key components: well array fabrication, membrane casting and transfer, filament design and placement, and driving circuitry to activate the sensor. The overarching goal of the research was to produce cost-effective, efficient, and robust membranes and filaments with a low-enough activation energy to allow for an implantable or wearable circuit design. Below, we describe the process by which this goal was achieved for each of the components.

### 2.1. Well Fabrication

As it is biocompatible, the sensing wells were made from Sylgard 184 Silicone Elastomer Polydimethylsiloxane (PDMS) [41]. The uncured PDMS was poured into molds that were printed on a Formlabs Form 3B stereo lithography printer using Formlabs LFBMCL01 Biomed Clear resin. The PDMS was deaerated in a vacuum chamber for 1 h and then cured in an oven at 65 °C for 5 h. After curing the PDMS, a square array of biopsy punches was used to stamp the sensing wells into the cured PDMS at a distance between wells of 1.3 cm well center to well center.

### 2.2. Membrane Fabrication and Transfer

**Figure 1:**
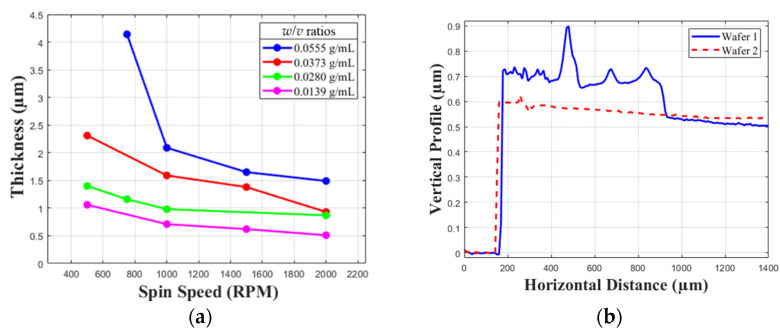
Nitrocellulose membrane and spin-coating data. (**a**) Nitrocellulose spin-coating curves for different solution weight/volume (*w*/*v*) ratios [36]. (**b**) Nitrocellulose membrane thickness profile for Wafer 1 (6-inch wafer) and Wafer 2 (3-inch wafer).

The membrane was constructed using a nitrocellulose thin film. As mentioned in the introduction, explosive nitrocellulose has a decomposition temperature between 55 °C and 130 °C [40]. This allows for a nitrocellulose membrane to be decomposed with a small amount of energy. Nitrocellulose is also easy to form into a film due to its solubility in acetone [42], which allows for spin coating of the acetone-nitrocellulose solution. Figure 1 shows characterization of nitrocellulose film thickness as a function of weight in solution and rotation speed [36] as well as achieved film profiles.

With a goal of obtaining a film thickness between 1 and 1.5 μm, in an effort to minimize activation energy, a 0.0139 g/mL solution of nitrocellulose in acetone was prepared by dissolving one half sheet of Thermo Scientific 88013 nitrocellulose membranes in 20 mL of 100% acetone in accordance with the spin curves in Figure 1. The solution was applied to the substrates at a spin speed of 2000 RPM. A suitable substrate for spin coating of the membranes required low adhesion to allow for membrane transfer to the well structure. The first attempt at membrane fabrication used a 2-inch sapphire wafer coated with Ti (14 nm)/Pt (100 nm) as the substrate. Platinum-coated substrates did not yield successful membrane transfer for the submicron thicknesses due to adhesion of the membrane to the platinum-coated substrate exceeding the force required to rupture the membranes.

To overcome the challenge of submicron membrane adhesion to the spin-coating substrate, the same spin speed and solution were used to spin coat onto a silicon wafer that was polished with microfiber cloths to smooth an applied coat of paraffin wax. To prepare the silicon wafer a microfiber cloth was used to rub a room temperature paraffin wax block and to rub the paraffin wax onto the wafer. Another cloth was used for polishing the wax layer until the silicon wafer appeared optically identical to its original appearance.

Membrane transfer was performed by applying a thin coat of uncured PDMS to the top of the cured PDMS well structure and placing it on the membrane to cure for twenty-four hours. Following twenty-four hours of curing, the membrane lifted up with the well structure. Minimal application of force to the well structure was required.

### 2.3. Filament Design and Construction

**Figure 2:**
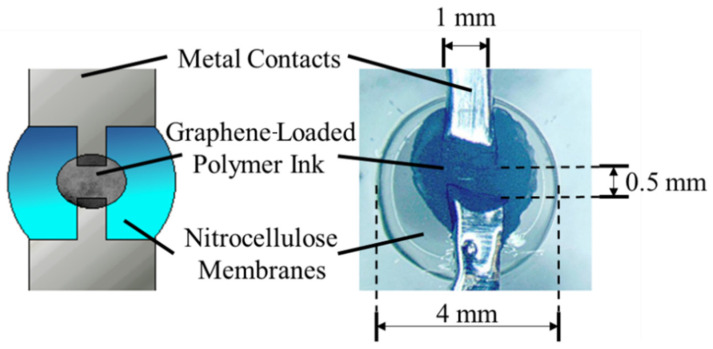
Graphene-loaded polymer filament structure.

Instead of an electron beam or thermal evaporation to deposit a thin metal filament that could be altered in thickness to concentrate power dissipation as performed in [37], a multi-material solution was developed that allowed for cost-effective and mechanically robust construction. Electrical contacts to deliver power to the well were made from metallic adhesive tape. However, contact could be provided by any method of metal deposition in the future, including stamped metallic foil affixed by PDMS, metallic adhesive tape, sputtering, or evaporation. The important component is that the metal portion of the contact was not continuous across the sensor well. Instead, graphene-loaded PEDOT:PSS conductive polymer ink was used in the 0.5 mm gap to provide a high-resistance connection directly over the sensor well. Graphene concentrations of 2.5 mg/mL and 5 mg/mL were tested. Application was performed by placing a drop of graphene-loaded polymer over the conductor gap and allowing it to dry. This process was repeated until the desired resistance was achieved. The resulting structure is shown in Figure 2.

### 2.4. Pulse Current Circuit

**Figure 3:**
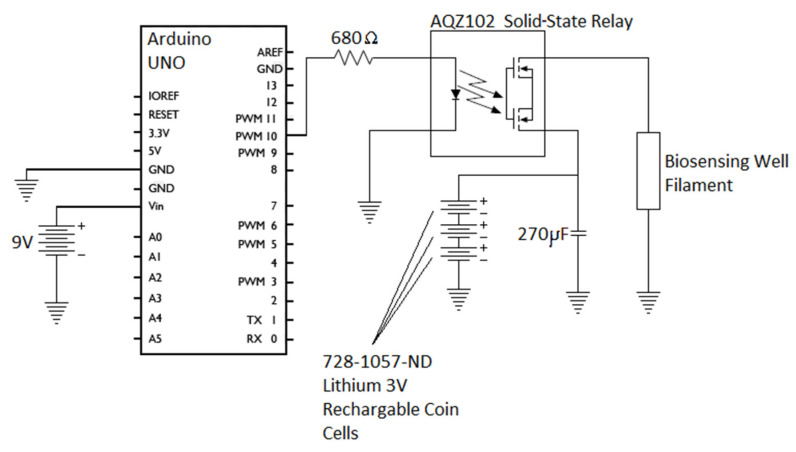
Pulse current circuit.

To activate the sensor well, a pulse current was applied to the filament using three, 728-1057-ND, 3 V lithium coin cells in parallel with a 270 µF capacitor, as shown in Figure 3. The circuit was activated via a solid-state relay controlled by the digital output of an Arduino UNO. The initial pulse duration was one second. Pulse durations were modified to find the minimum possible energy required to activate the sensor well. Many microcontrollers currently on the market are capable of running on the rechargeable 3-volt lithium batteries used for well activation [43]. This would allow the same batteries being used for activation of the sensing well to power the control circuitry as well.

### 2.5. Assembly

**Figure 4:**
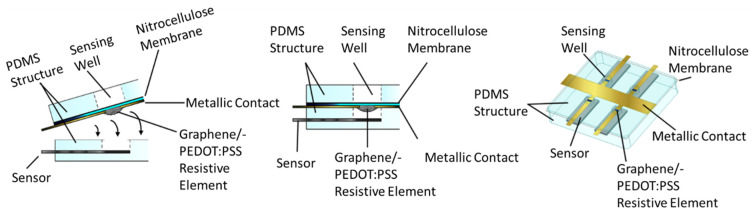
Assembled sensor well, membrane, and filament.

The complete fabricated assembly can be seen in Figure 4. This structure is achieved by first applying a thin coat of uncured PDMS to the nitrocellulose membrane and conductors lying on the PDMS well structure without coating the nitrocellulose and filament directly over the wells themselves. This is then placed firmly onto a PDMS structure that houses a glucose sensing strip. Enough pressure must be applied to evacuate all air between the two PDMS fixtures. The PDMS is allowed to cure for twenty-four hours. A strip was used because the primary focus of investigation is on-demand well activation for any electrochemical sensor. A fully integrated system may be explored in the future.

By combining the two portions with the filament facing the sensor, the filament is protected from the fluid until the well is activated. Protecting the filament is necessary to ensure the lifespan of the device as well as to minimize the amount of power needed to activate the sensor. Since the thermal and electrical conductivity of the biofluid is likely higher due to its higher water content than that of the air under the membrane, it would require more energy to activate the membrane if the filament were immersed in the biofluid [44].

### 2.6. Test and Measurement

Various filament resistances, pulse durations, and activation voltages were used to achieve the minimum possible energy for sensor well activation. Filaments with resistances between 10^4^ Ω and 10^2^ Ω were tested with voltages of either 9 V (3 Lithium cells) or 18 V (6 Lithium cells). Pulse durations ranged from 50 ms to 1000 ms. Resistance was varied by difference in percent graphene loading in PEDOT:PSS and the number of drops applied to the metal contacts, which increased the filament thickness. Pulse durations were controlled by a variable in the Arduino’s firmware and voltage was controlled by the number of in-series batteries powering the sensor well filament. The status of membrane decomposition or bursting (sensor activation) was recorded following pulse application.

## 3. Results

Membranes that would be transferred onto various sensor wells were spin coated on to two different silicon wafers, one 3 inches and one 6 inches, using a 0.0139 g/mL solution of nitrocellulose in acetone at a spin rate of 2000 RPM as mentioned in “Membrane Fabrication and Transfer”. A Dektak 150 Stylus Profiler was used to measure the film profile compared to the substrate, to obtain results for membrane thickness and uniformity. The film thickness profiles for the two wafers are shown in Figure 1. At the edge of where the membrane is lifted from the substrate, there are edge effects that cause the profile to be distorted. Thickness of the film is based on data in the bulk material following the distorted edge, in order to obtain an accurate reading. To ensure that an accurate measurement was obtained, the horizontal distance of the profiler was set such that membrane profile would have a sufficient stable region of measurement. Where the membrane thickness stabilizes, it can be seen that the film thickness stabilizes below 600 nm, which falls within a range of 0.5 to 1 micro-meter. This meets the original goal of submicron thickness. The film profiles show no large deformation or porosity, nor was any seen under the microscope. Membrane transfers were conducted successfully onto seven four-well array structures from paraffin wax-polished silicon. Five of the structures were transferred from the 6-inch wafer and two were transferred from the 3-inch wafer.

After membranes were placed on the wells, filaments were constructed with two different concentrations of graphene loading on the sensor well membranes, with two or three drops of graphene-loaded polymer applied. Variables were modified to determine the lowest possible energy required to activate the sensing well. Data for each tested filament can be found in Table 1. The graphene-loaded polymer ink eventually reached a point at which the resistance could not be significantly decreased with additional drops. This was because lateral spread of the ink prevented an increase in filament thickness. The lowest resistance that could be obtained with this mixture averaged 108 Ω. Of the wells that had filaments with resistances in the lowest order of magnitude, the smallest pulse duration needed to activate the well was 85 ms at 9.04 V. The resulting pulse energy was 68 mJ, which is comparable with the average activation energy values obtained in prior work, which used an electron beam or thermal evaporation to manufacture filaments [37].

**Figure 5:**
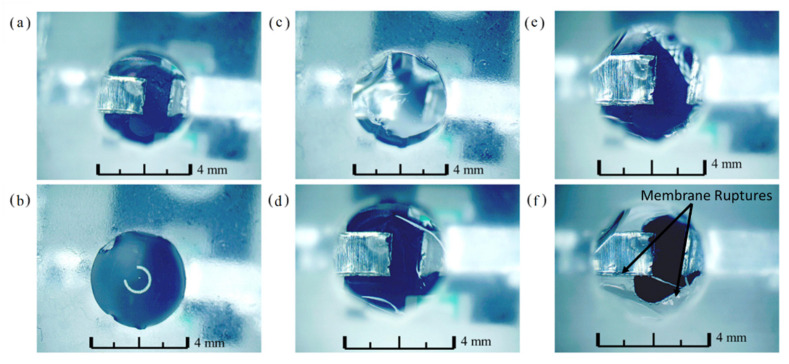
Well activation with simulated ISF (45× Optical Magnification): (**a**) Top of dry well prior to filling; (**b**) Well above membrane filled with water before activation; (**c**) Well immediately after activation; (**d**) Well thirty seconds post-activation; (**e**) Well two minutes post-activation; (**f**) Well with sensing strip removed.

One well was filled with simulated ISF prepared using [45] to determine if the presence of liquid on top of the well membrane would prevent activation. The fluid was not in contact with the filament, however, with the membrane thickness being roughly 600 nm there was a concern that heat dissipation into the fluid could be an issue. Pictures were taken before filling the well and after filling the well, as seen in Figure 5a,b. After well activation, a photo was taken immediately and thirty seconds later, as shown in Figure 5c,d. Two minutes after activation, all of the liquid had drained into the sensing portion of the well through the activated membrane through rupture points. This can be seen in Figure 5e. After two minutes, the sensor was removed from the well to obtain a clear image of the breaks in the activated well.

**Figure 6:**
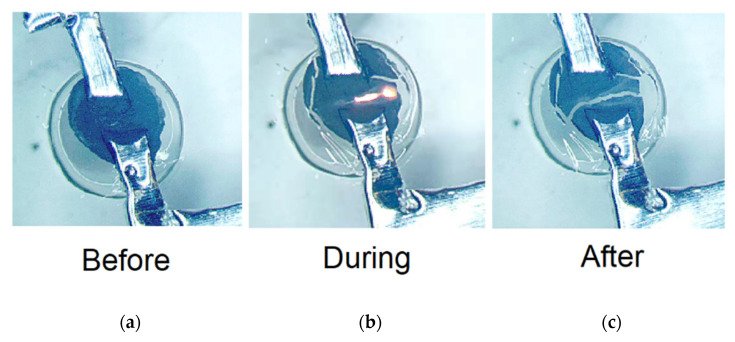
Explosive decomposition of well membrane (45× optical magnification) (**a**) before activation; (**b**) during activation; (**c**) after activation.

The activated well shown in Figure 6 corresponds to the fourth row of data in Table 1. Images were taken before, during, and after activation to verify explosive decomposition of the nitrocellulose membrane. Finally, impermeability of the submicron nitrocellulose membrane was tested by submerging the membrane-sealed PDMS wells with blue food color in water for three weeks. No indication of water leak into the PDMS wells was detected. This is consistent with microscopic observations of membrane integrity and no indication of porosity.

## 4. Discussion

The data show that sensor well activation can occur in the high tens of millijoules energy range. This is similar to results obtained in previous data in which an electron beam and thermal deposition were used for filament application [36,37,38]. Electron beam and thermal evaporation, as well as sputtering, are costly thin metal deposition methods. In contrast, the use of a material such as the graphene-infused polymer across two conductors merely requires a CNC pipetting system to apply the material between the contacts on the sensor well. Metal deposition in this case could potentially use laser cut contacts applied with PDMS as an adhesive, inkjet printed contacts, or stamped foil contacts. These systems are an order of magnitude less expensive and produce metal structures that are less likely to crack due to their thickness [46].

Relatively little care or attention to process detail was required to successfully transfer submicron-thick nitrocellulose films to PDMS once paraffin wax-polished silicon wafers were used as the substrate. This realization led to an easy and low-cost process that was tolerant to human factors in execution. This was evidenced by the successful transfer of several submicron nitrocellulose membranes. No submicron-thick membranes were successfully transferred using platinum-coated wafers.

The data in Table 1 show that the well could be routinely activated with as little as 68 mJ of energy. The low energy required for decomposition of the nitrocellulose membrane was provided by three series 3 V lithium coin cells all in parallel with a 0.27 mF capacitor, for all wells activated with approximately 9 V. The coin cells had a diameter of 6.75 mm and a thickness of 2.36 mm. This circuit can work with a smaller, lower-powered micro-controller. This would allow for a circuit that could fit in dimensions constrained only by the size of the batteries and the size of the sensor array, and could be manufactured on a single printed circuit board (PCB). Long-lasting CGMs with rechargeable batteries may be able to last up to nearly four years with a 100-sensor array and could potentially be implanted using these small batteries and a smaller micro-controller, allowing the patient to live free of a device attached to the outside of their bodies.

Future work will focus on several areas of potential improvement. Using a different polymer for the filament may lead to lower voltages required for activation by reducing resistance and allowing for a smaller number of batteries in the circuit. The major limitation to reduction in resistance was lateral spread of the polymer ink. A higher-viscosity polymer or potentially carbon ink jetting could be used to reduce resistance by allowing for thicker filaments to be deposited. Adjustment of graphene loading is an option, however, the properties of the ink (viscosity, adhesion, drop size, etc.) appear to change significantly with higher loading and the controllability of resistance begins to become more difficult. If the resistance is too low, then enough heat may not be generated in a small enough area to activate the nitrocellulose membranes. By reducing the resistance at least one order of magnitude, it is possible that the energy needed to decompose the membrane could be reduced by an order of magnitude as well. The device could benefit from an energetic polymer membrane with lower activation energy than that of nitrocellulose. A lower activation energy could result in even greater miniaturization of the support circuitry due to a lower energy required for decomposition. Future work should also look into the potential biocompatibility of nitrocellulose for implantation. Thermal decomposition of nitrocellulose [C_6_H_7_(NO_2_)3O_5_]_n_ produces water and gaseous products according to the following reaction:

2 × [C_6_H_7_(NO_2_)_3_O_5_] ≥ 7H_2_O + 7CO_2_ + CO + 3N_2_ + 4C

Complete decomposition of a 1 μm nitrocellulose membrane of 1 mm × 1 mm lateral size will produce ~0.75 μL mixture of CO_2_, CO, and N_2_ (calculated for 37 °C and assuming all water is produced as liquid). A 0.47 μL measure of CO_2_ produced will readily dissolve in ISF, and the rest, 0.28 μL of CO + N_2_ mixture, will dissolve slowly and spread by the blood stream. When partial decomposition of the membrane, smaller actual thickness, and diffusion-limited exchange between ISF and blood are taken into consideration, these levels are too insignificant to cause any negative effects such as blockage of blood vessels. Moreover, at physiological concentrations, CO was shown to mediate signaling processes only in the brain, liver, and endothelium [47], which are not targeted for implantation of glucose sensors. Therefore, any potential effects of CO signaling are negligible. Given a density of 1.71 g/cm^3^, this same membrane would have a mass of 1.71 μg [48]. The heat of combustion for nitrocellulose in the 13.3% nitrous group is approximately 3630 J/g [49]. This results in an additional 6.2 mJ of energy dissipated in the process of combustion, a value of less than 10% of the energy used to disintegrate the membrane. Given the 1 mm air gap between the membrane and sensor and the high thermal conductivity of the water containing ISF above the membrane compared to the air below, the impact of additional energy on the sensor is negligible.

## 5. Conclusions

In this work, we have demonstrated an easy to fabricate, mechanically robust, and cost-effective method for on-demand activation of biosensors. A paraffin wax-polished silicon wafer allows for ease of removal and transfer of a spin-coated, submicron-thick nitrocellulose membrane. This nitrocellulose membrane has been found capable of protecting a biosensing well and can be activated on demand by a pulse current with an energy as low as 68 mJ. Use of a conductive-polymer filament connecting contacts over the well allowed for fabrication suitable for mass production without the need for any expensive equipment such as an electron beam/thermal evaporator or sputtering system. Further work would result in more miniature devices allowing for use in on-body or implanted CGMs. With larger arrays, the lifetime of the device can be extended by more than an order of magnitude. For these reasons, this technology is an ideal candidate for helping to increase patient adoption of CGMs and lower the cost of treatment over time.

## Figures and Tables

**Table 1:** Pulse energy data and activation status as a function of graphene loading, number of drops, pulse duration, applied voltage, and filament resistance.

Graphene Loading (mg/mL)	Number of Drops	Voltage (V)	Resistance (Ω)	Pulse Duration (ms)	Well Ruptured (Yes/No)	Pulse Energy (mJ)
5	3	9.14	112	1000	Yes	746
2.5	3	17.99	1093	1000	Yes	296
5	3	8.98	108	150	Yes	112
5	4	9.01	102	100	Yes	80
5 *	3	9.01	112	100	Yes	72
5	3	9.04	102	85	Yes	68
5	3	8.95	113	75	No	53
5	3	9.00	105	50	No	39
2.5	2	18.00	17,800	1000	No	18.2

* Well ruptured with simulated ISF above well at room temperature.

## Data Availability

Data may be obtained via email to lundquistjd@vcu.edu or etopsakal@vcu.edu.
